# Enhancement of collagen deposition and cross-linking by coupling lysyl oxidase with bone morphogenetic protein-1 and its application in tissue engineering

**DOI:** 10.1038/s41598-018-29236-6

**Published:** 2018-07-17

**Authors:** T. Rosell-Garcia, F. Rodriguez-Pascual

**Affiliations:** Centro de Biología Molecular “Severo Ochoa” Consejo Superior de Investigaciones Científicas (C.S.I.C.)/Universidad Autónoma de Madrid (Madrid), Madrid, Spain

## Abstract

Cultured cell-derived extracellular matrices (ECM)-based biomaterials exploit the inherent capacity of cells to create highly sophisticated supramolecular assemblies. However, standard cell culture conditions are far from ideal given the fact that the diluted microenvironment does not favor the production of ECM components, a circumstance particularly relevant for collagen. An incomplete conversion of procollagen by C-proteinase/bone morphogenetic protein 1 (BMP1) has been proposed to severely limit *in vitro* collagen deposition. BMP1 also catalyzes the proteolytic activation of the precursor of the collagen cross-linking enzyme, lysyl oxidase (LOX) to yield the active form, suggesting a deficit in cross-linking activity under standard conditions. We hypothesized that the implementation of fibroblast cultures with LOX and BMP1 may be an effective way to increase collagen deposition. To test it, we have generated stable cell lines overexpressing LOX and BMP1 and studied the effect of supernatants enriched in LOX and BMP1 on collagen synthesis and deposition from fibroblasts. Herein, we demonstrate that the supplementation with LOX and BMP1 strongly increased the deposition of collagen onto the insoluble matrix at the expense of the soluble fraction in the extracellular medium. Using decellularization protocols, we also show that fibroblast-derived matrices regulate adipogenic and osteogenic differentiation of human mesenchymal stem cells (MSC), and this effect was modulated by LOX/BMP1. Collectively, these data demonstrate that we have developed a convenient protocol to enhance the capacity of *in vitro* cell cultures to deposit collagen in the ECM, representing this approach a promising technology for application in tissue engineering.

## Introduction

The extracellular matrix (ECM) is a dynamic microenvironment that importantly influences a number of cellular processes, including cell proliferation, adhesion, migration, and differentiation, as well as plays key roles in homeostasis and regeneration of tissues and organs^1^. Tissue engineering has exploited these properties and ECM-based biomaterials are today more than a promising therapy for tissue repair and regeneration^2,3^. The use of native ECM substrates over artificially assembled ECM scaffolds allows for better preservation of the appropriate cell growth microenvironment, thereby speeding up the repair of damaged tissue^4^. Therefore, there remains a strong interest in developing techniques and protocols to enhance the innate capacity of cells to create their own ECM *in vitro*. However, despite significant progresses, optimal conditions for rapid and efficient deposition of ECM components, mainly collagen, the most important structural biomolecule, are still missing. This behavior has been attributed to the diluted culture media that severely limit extracellular post-translational modifications of collagen, namely the cleavage of the C propeptide, a reaction catalyzed by C-proteinase/bone morphogenetic protein 1 (BMP1), and the formation of covalent cross-links, initiated by members of the lysyl oxidase (LOX) family^5,6^. These enzymatic activities are intimately intricate as BMP1 also catalyzes the proteolytic activation of the precursor of LOX to yield the active form^7^.

Several approaches have been developed to facilitate collagen deposition in standard cell culture conditions, including ascorbic acid and serum supplementation^8^. One interesting approach was designed based on the addition of inert macromolecules in the culture media in order to imitate a dense extracellular space, a biophysical phenomenon known as macromolecular crowding^5,9^. Under this principle, the addition of dextran sulfate (DxS) or Ficoll^TM^ has been reported to enhance the capacity of a number of different cell cultures, including fibroblasts, keratinocytes, tenocytes, chondrocytes or mesenchymal stem cells, to deposit abundant extracellular matrix^10–15^.

Addressing the cause root of the problem, we propose the hypothesis that the addition of LOX and BMP1 may represent a strategy to boost *in vitro* deposition of collagen. Here we show that implementing fibroblast cultures with supernatants enriched in LOX and BMP1 from stable HEK293 cell lines strongly increased the deposition of collagen onto the insoluble matrix at the expense of the soluble fraction in the extracellular medium. Using decellularization protocols, we also provide evidence that fibroblast-derived matrices are able to regulate the adipogenic and osteogenic differentiation of human mesenchymal stem cells (MSC), a powerful cell tool in regenerative medicine, being this effect modulated by LOX/BMP1. Our study demonstrates this system is very effective to enhance the capacity of cell cultures to promote collagen deposition, making this technology a promising approach for application in tissue engineering where a demand exists for a fast and quantitative collagen deposition.

## Results

### *In vitro* collagen deposition is slow and has low efficiency

**Figure 1:**
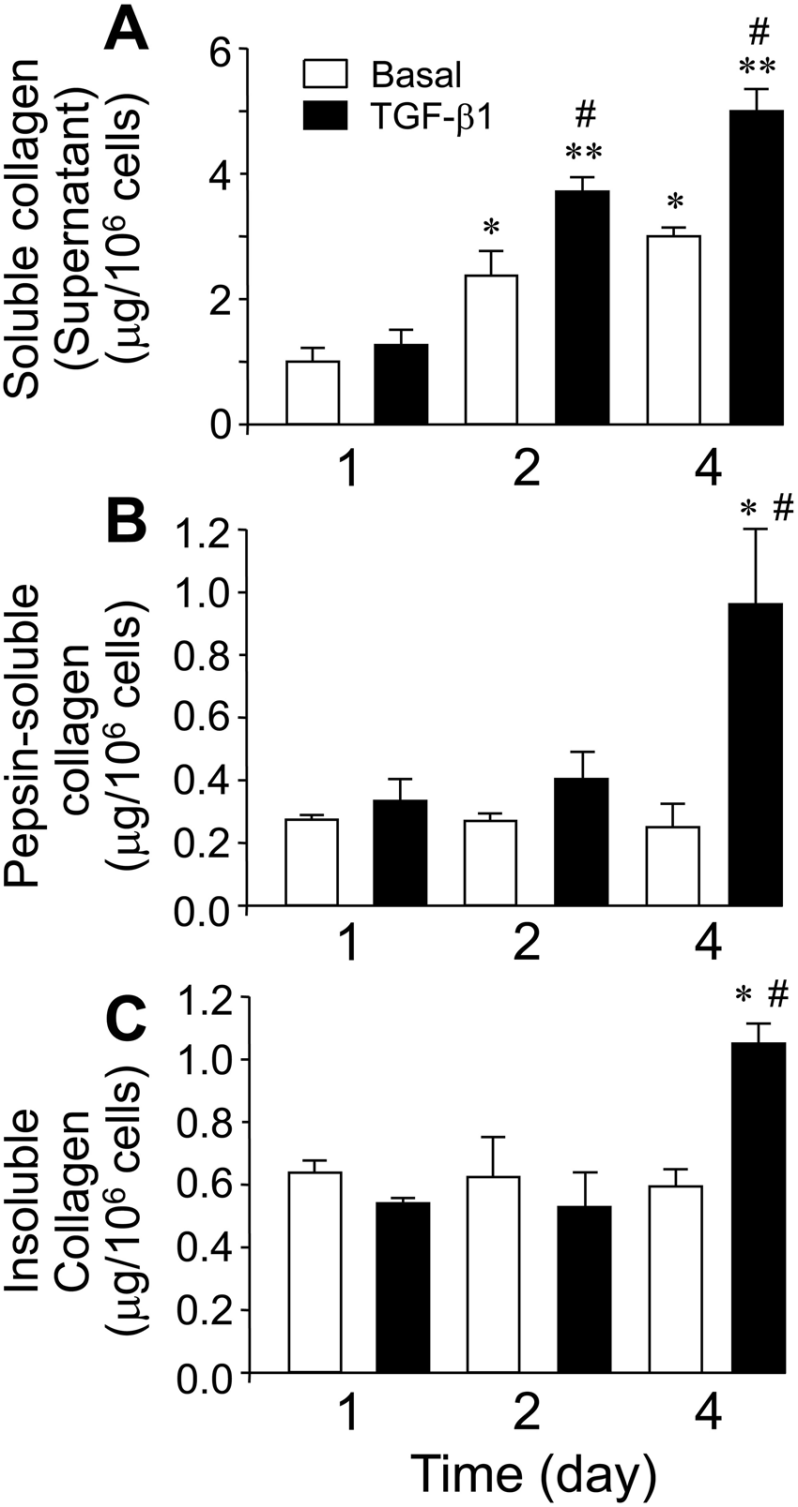
Time-dependent stimulation of collagen synthesis and deposition in fibroblasts incubated with and without TGF-β1. (**A**) Soluble collagen in the supernatant. (**B)** Pepsin-solubilized collagen fraction associated to cell monolayer. (**C)** Insoluble collagen deposited into the matrix. Collagen fractions were determined from cells incubated for 1 to 4 days in the absence (white bars) or presence of 5 ng/ml TGF-β1 (black bars) as described under Materials and Methods. Values are represented as μg collagen per million of cells (mean ± SEM, n = 6; *P < 0.05 or **P < 0.01vs one day in the absence of TGF-β1, and ^#^P < 0.05 vs the corresponding time value in the absence of TGF-β1).

We have studied the synthesis and deposition of collagen in cultures of human lung fibroblast (CCD19-Lu) cells under basal conditions or incubated with the profibrotic cytokine transforming growth factor (TGF)-β1 for time periods ranging from one to four days **(**Fig. 1**)**. As shown in Fig. 1A, the levels of the soluble form of secreted collagen progressively accumulated in cell supernatants from fibroblasts incubated under basal conditions, and this effect was further augmented in cells stimulated with TGF-β1. Deposition into the matrix as pepsin-soluble or insoluble forms only modestly increased in cells incubated for four days with TGF-β1 (Fig. 1B,C). Acid-based buffer solubilized negligible amounts of collagen, indicating this pool is not stable in our experimental conditions (data not shown). Overall, these results indicate that, despite an active production and secretion of collagen precursors, *in vitro* deposition is an unfavoured process.

### Generation of HEK293 cell lines overexpressing lysyl oxidase (LOX) and bone morphogenetic protein-1 (BMP1)

**Figure 2:**
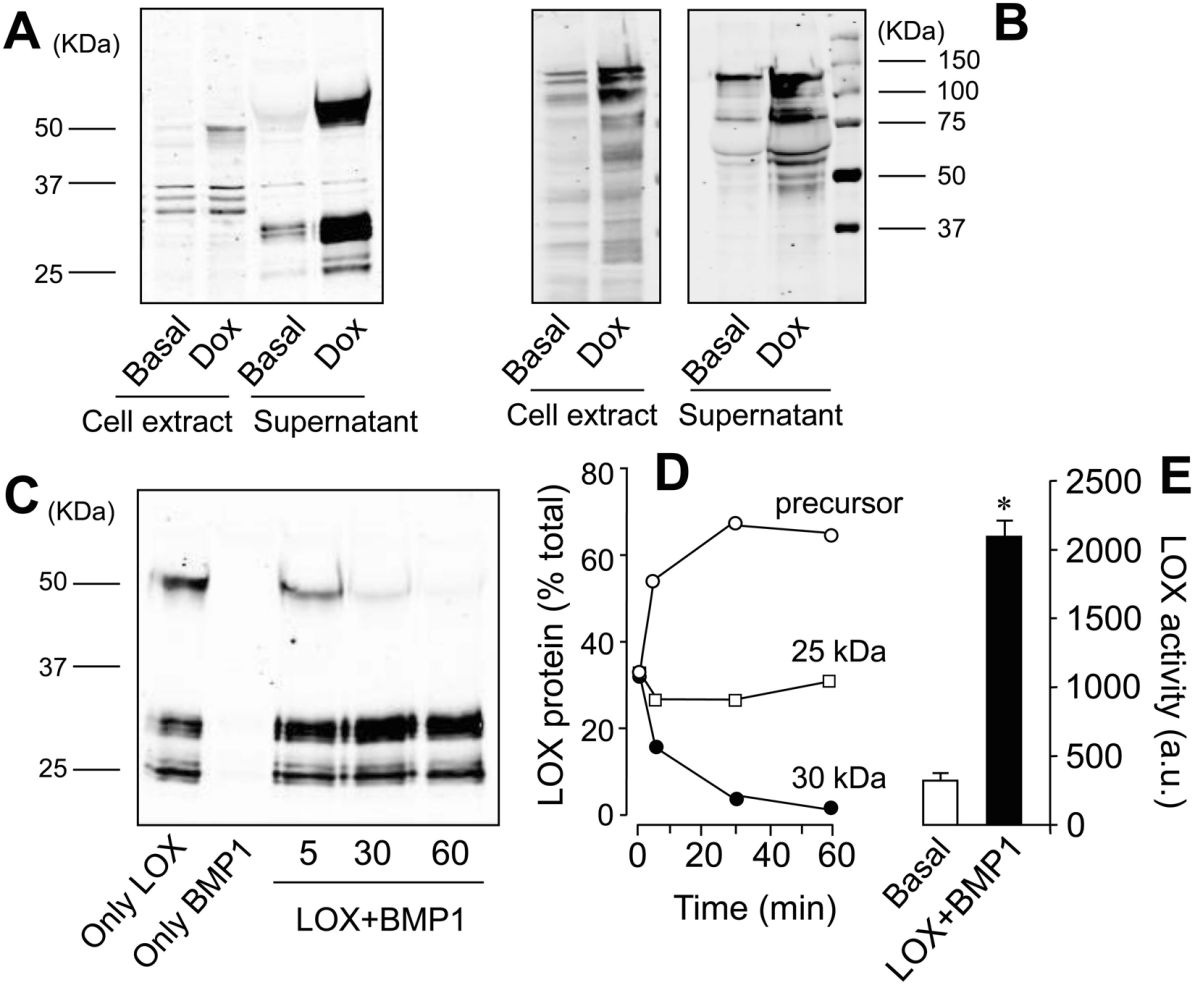
Generation of HEK293 cells overexpressing secreted and active forms of LOX and BMP1 proteins. Induction of LOX (**A**) and BMP1 (**B**) proteins in HEK293 cells upon incubation with the tetracycline analog, doxycycline (Dox), at 10 μM as assessed by western blotting using total cell extracts or Amicon-concentrated aliquots of the cell supernatants. (**C**) Combination of cell supernatants containing LOX and BMP1 proteins gives rise to the proteolytic activation of LOX as assessed by western blotting. The blot shown correspond to a representative experiment performed twice with two independent preparations. (**D**) LOX-immunoreactive bands from results shown in panel (**C**) were quantified and expressed as percentage of total: 50 KDa precursor (closed circle), 30 KDa active form (open circle), and 25 KDa unknown band (open squares). (**E**) LOX enzymatic activity as measured using Amplex red assay in cell supernatants from uninduced cells (Basal, white bar) or induced with Dox and incubated with BMP1 for 60 min (LOX + BMP1, closed bars). Values are represented as arbitrary fluorescent units (mean ± SEM, n = 6; *P < 0.01).

Several evidences in the literature suggest that low activity levels of BMP1 and LOX enzymes significantly limit collagen deposition *in vitro*^5,6^. To validate the addition of LOX and/or BMP1 as an effective strategy to boost *in vitro* deposition of collagen, we generated HEK293 cell clones stably expressing LOX and BMP1 constructs under tetracyclin-dependent control. As shown in Fig. 2A, LOX transfectants expressed and secreted to the extracellular medium several LOX immunoreactive bands including the precursor of about 50 KDa, and shorter bands of 25 and 30 KDa. In a similar approach, BMP1 transfectants showed doxycycline-sensitive expression and secretion of a complex mixture of BMP1 forms ranging from 60–100 KDa, likely representing precursor and processed forms **(**Fig. 2B**)**. The presence of the 50 KDa band in supernatants from LOX-overexpressing cells indicates a limited capacity to process and activate the enzyme. Interestingly, incubation of cell supernatants containing LOX with those with BMP1 promoted the proteolysis of the precursor pro-LOX to the active form of 30 KDa in a time-dependent manner (Fig. 2C,D). The shortest LOX form of 25 KDa was not modified by the action of BMP1 and seems to result from the action of a protease different from BMP1. LOX enzymatic activity was assessed in a fluorometric assay using supernatants from basal and doxycycline-incubated cells. As shown in Fig. 2E, the induction of the expression of LOX together with the combination with BMP1 supernatants promoted a strong increase in LOX enzymatic activity. Taken together, we succeed in generating HEK293-based cell systems to produce supernatants enriched with LOX and BMP1 enzymes which, when combined together, recapitulated *in vitr*o the proteolytic activation of LOX.

### Addition of recombinant lysyl oxidase (LOX) and bone morphogenetic protein-1 (BMP1) strongly increases collagen deposition *in vitro*

**Figure 3:**
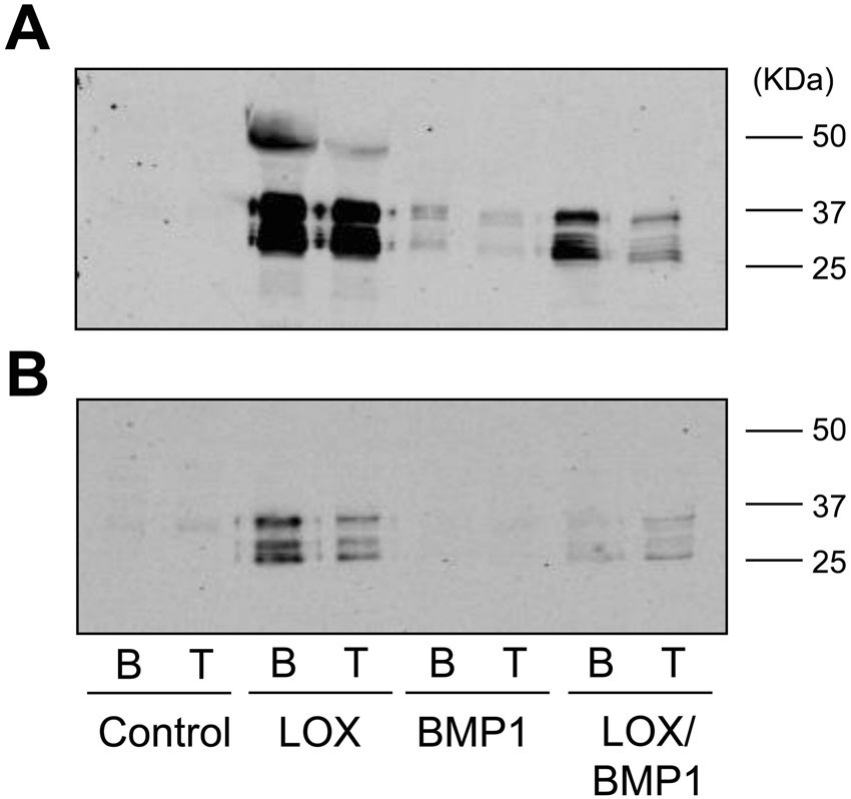
LOX immunoreactivity in the supernatants of fibroblast cultures supplemented with LOX- and BMP1-containing conditioned media. LOX, BMP1 or LOX/BMP1 supernatants were added to fibroblast cultures in the presence (T) or absence (basal, **B**) of TGF-β1 and LOX immunoreactivity assessed by western blotting at the beginning of the experiment (**A**), one day) or at the end (**B**), four days). The blots shown correspond to representative experiments performed twice with two independent preparations.

**Figure 4:**
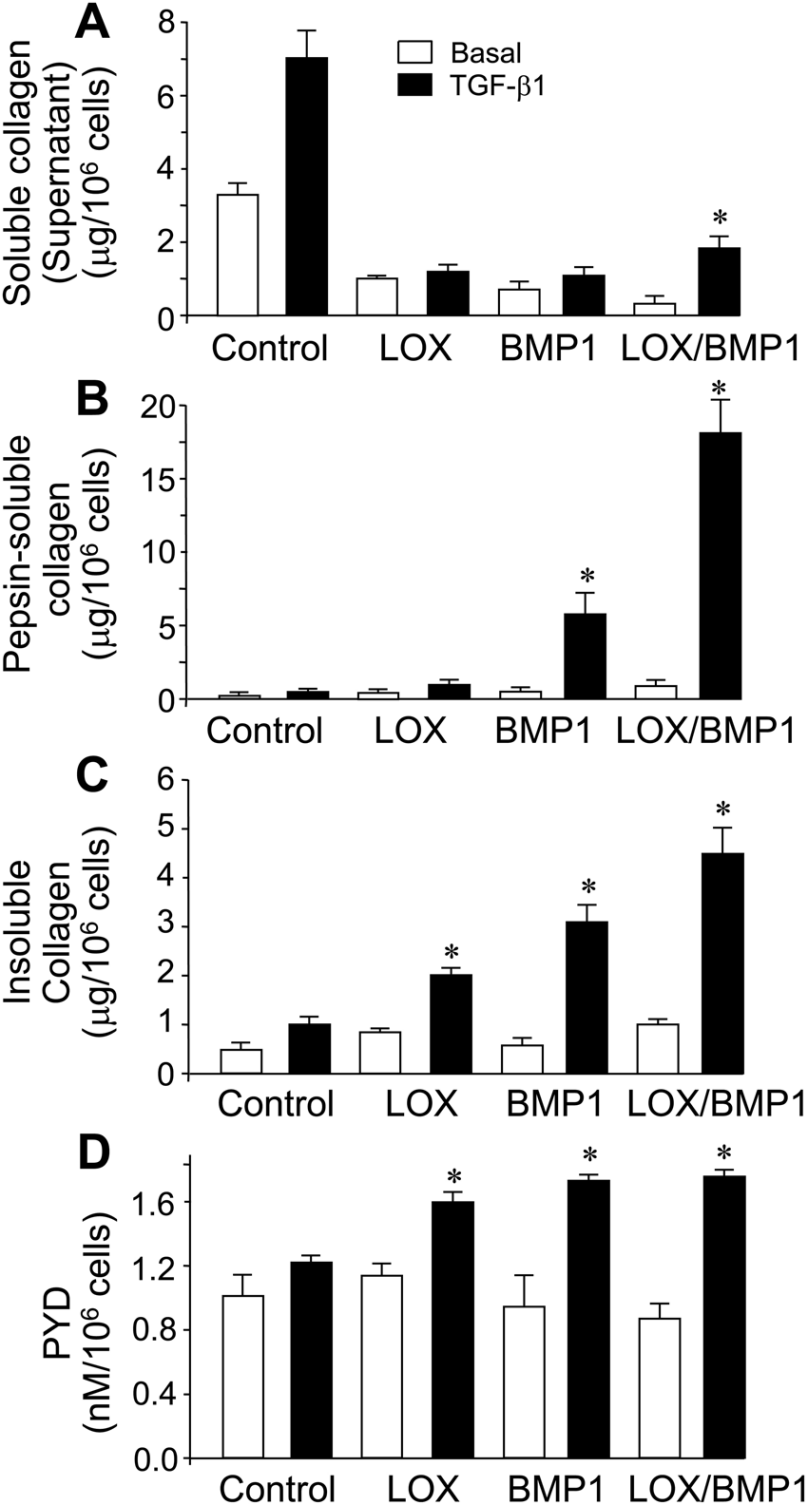
Effect of the supplementation with LOX/BMP1 supernatants on collagen deposition from fibroblast cultures. Collagen fractions as measured in Fig. 1 were analyzed in fibroblasts exposed to conditioned media from control or LOX- and BMP1-overexpressing cells and incubated with and without TGF-β1 for 4 days. (**A**) Soluble collagen in the supernatant. (**B**) Pepsin-solubilized collagen fraction associated to cell monolayer. (**C**) Insoluble collagen deposited into the matrix. (**D**) LOX-derived pyridinoline (PYD) cross-link levels in the deposited matrix from fibroblast cultures exposed to conditioned media as assessed by specific ELISA. Values are represented as μg collagen or concentration of PYD per million of cells (mean ± SEM, n = 6; *P < 0.05 vs the corresponding control values with TGF-β1).

We have first checked the proteolytic activation of LOX in fibroblasts exposed to supernatants. As shown in Fig. 3A, fibroblasts incubated for one day with only LOX supernatants displayed a significant amount of the unprocessed LOX precursor, again indicating a limited cell capacity to *in vitro* process the proenzyme. In contrast, the combination of recombinant LOX and BMP1 resulted in complete proteolysis of the pro-LOX. The presence of processed forms of LOX in fibroblasts incubated with BMP1 alone indicated that the protease promoted the processing of endogenously produced LOX. No detectable LOX bands were observed in fibroblasts exposed to control media. After four days of incubation with supernatants, proteolytic conversion of pro-LOX enzyme was complete, even in the absence of added BMP1 (Fig. 3B). Interestingly, LOX immunoreactive signals were lower in supernatants from LOX/BMP1 than those from only LOX (at both one and four days), as well as in LOX (or LOX/BMP1) at one day compared to corresponding samples at four days, indicating that as soon as the processed forms of LOX are generated, they are either degraded or retained into the matrix. We have then studied the effect of these supernatants on collagen synthesis and deposition. As shown in Fig. 4A, as opposed to cells exposed to control media, the incubation of fibroblasts with cell supernatants containing either LOX, BMP1 or a mixture of both abrogated the accumulation of soluble collagen in the extracellular medium, both in the absence or presence of TGF-β1, an effect that was further corroborated by immunoblotting using an anti-col1α1 antibody (Suppl. Fig. 1). Concomitantly with this drastic reduction, both pepsin-soluble and -insoluble fractions from TGF-β1-treated cells were found to significantly increase, being higher in cells incubated with the mixture of LOX/BMP1 than with either only LOX or BMP1, an observation that suggests a synergic action for the effect of both enzymes (Fig. 4B,C). LOX enzyme catalyzes the oxidative deamination of telopeptide lysine/hydrolysine residues to yield highly reactive aldehydes that further react to form immature and then mature permanent cross-links^16,17^. The preferential use of hydroxylysine versus lysine in cross-linking reactions determines a distinctive pattern of maturational products, with higher levels of pyridinolines than of pyrroles, as is usually found in cartilage, bone or aorta^18^. Hydrolyzed pepsin-insoluble pellets were assayed with a specific ELISA for the presence of pyridinoline cross-links (PYD). As shown in Fig. 4D, compared with control, the exposure of fibroblasts to LOX and/or BMP1 supernatants promoted the formation of PYD cross-links, indicating that a significant part of the deposited collagen is formed through this maturation pathway.

**Figure 5:**
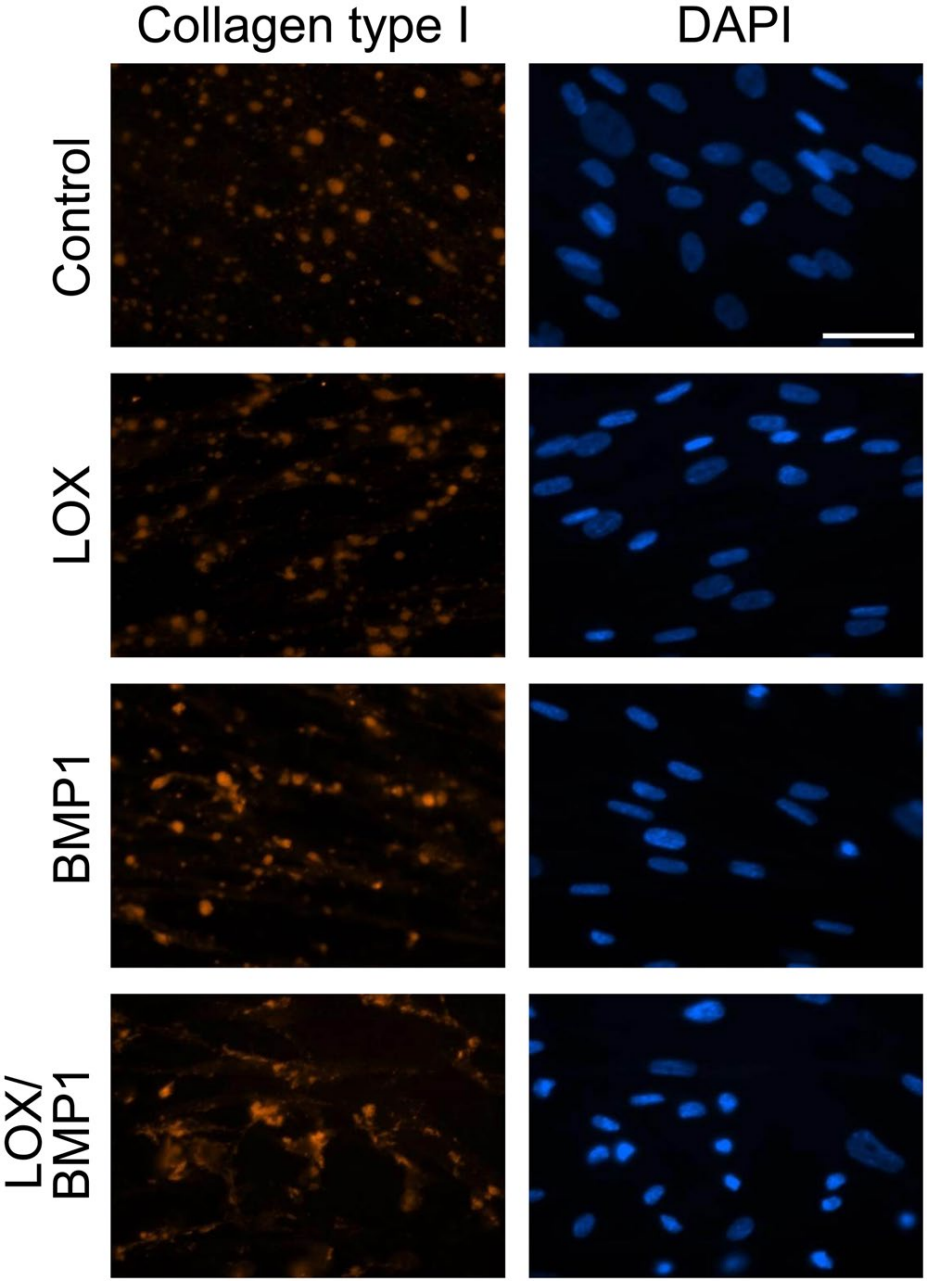
Immunofluorescence analysis of collagen type I deposition from fibroblast cultures exposed to LOX/BMP1 supernatants. Fibroblasts exposed to control or LOX/BMP1 supernatants and incubated in the presence of TGF-β1 for 4 days were processed for immunofluorescence analysis of collagen type I as described under Materials and Methods. Micrographs shown correspond to representative results of staining for collagen type I (red) and nuclei using DAPI (blue) performed twice with two independent preparations. Bars = 50 μm.

**Figure 6:**
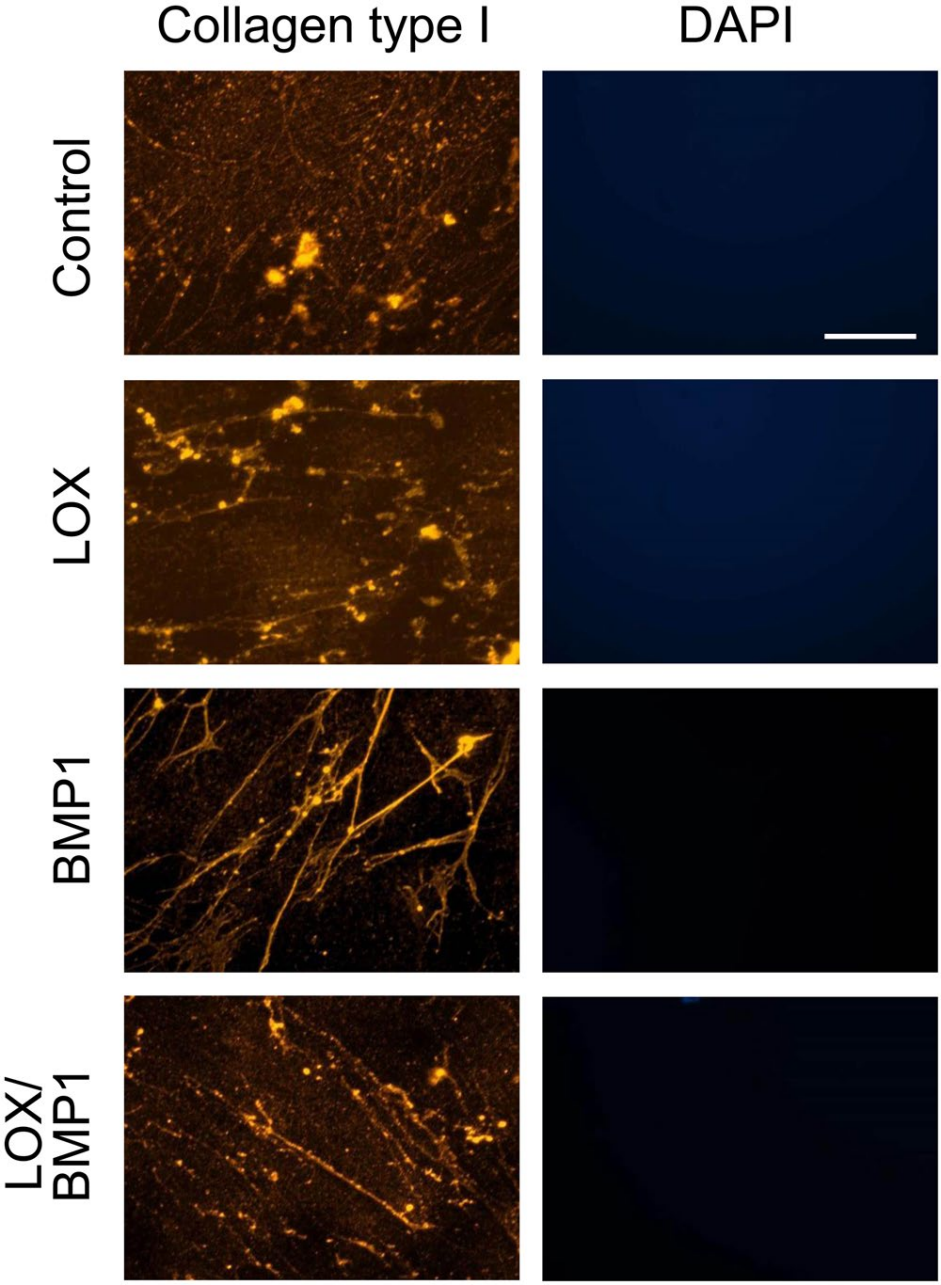
Immunofluorescence detection of deposited collagen I in decellularized matrices from fibroblasts exposed to LOX/BMP1 supernatants. Fibroblast monolayers exposed to control or LOX/BMP1 supernatants in the presence of TGF-β1 for 4 days were decellularized before processing for immunofluorescence analysis of collagen type I as described under Materials and Methods. Micrographs shown correspond to representative results of staining for collagen type I (red) performed twice with two independent preparations. Bars = 50 μm. The absence of DAPI staining confirmed the effectiveness of the decellularization procedure.

We have also analyzed the effect of LOX and BMP1-containing supernatants by immunofluorescence analysis using an anti-col1α1 antibody. As shown in Fig. 5, fibroblasts exposed to control media displayed collagen type I immunoreactivity in the form of small and large aggregates. While this appearance was not significantly modified by LOX supernatants, cells exposed to BMP1 and particularly to the mixture of BMP1 and LOX showed a more distinctive pattern of immunoreactivity that includes the presence of fibrous material, likely consistent with their deposition to the matrix, rather than associated with the cell layer. This was further corroborated with experiments in decellularized matrices. As shown in Fig. 6, upon removal of the cell-associated material, a more fibrous pattern was observed in deposited matrix from cells exposed to BMP1 and the mixture of BMP1 and LOX. DAPI staining confirmed that the extraction procedure efficiently removed the cell layer. Using decellularized matrices, we have also investigated whether the deposition of other matrix components is also increased under LOX/BMP1 supplementation. As shown in Suppl. Fig. 2, low but significant levels of collagen type IV in the form of small aggregates were observed in decellularized matrices from control fibroblasts, and these levels decreased in matrices from LOX and/or BMP1-treated fibroblasts. Collagen type III and fibronectin were also detected at very low levels in controls matrices, and found not to significantly change upon LOX/BMP1 treatments. The presence of LOX immunoreactivity in these matrices was also investigated by immunofluorescence. As shown in this figure, LOX was not detected throughout the experimental conditions, an observation suggesting LOX is not efficiently incorporated into the ECM.

Taken together, our results show that the implementation of fibroblast cultures with supernatants enriched in LOX and BMP1 was an effective approach to strongly increase the deposition of collagen type I onto the insoluble matrix, showing negligible effects on other matrix components.

### Fibroblast-derived matrix modified by lysyl oxidase (LOX) and bone morphogenetic protein-1 (BMP1) regulates the differentiation of human mesenchymal stem cells (MSC)

**Figure 7:**
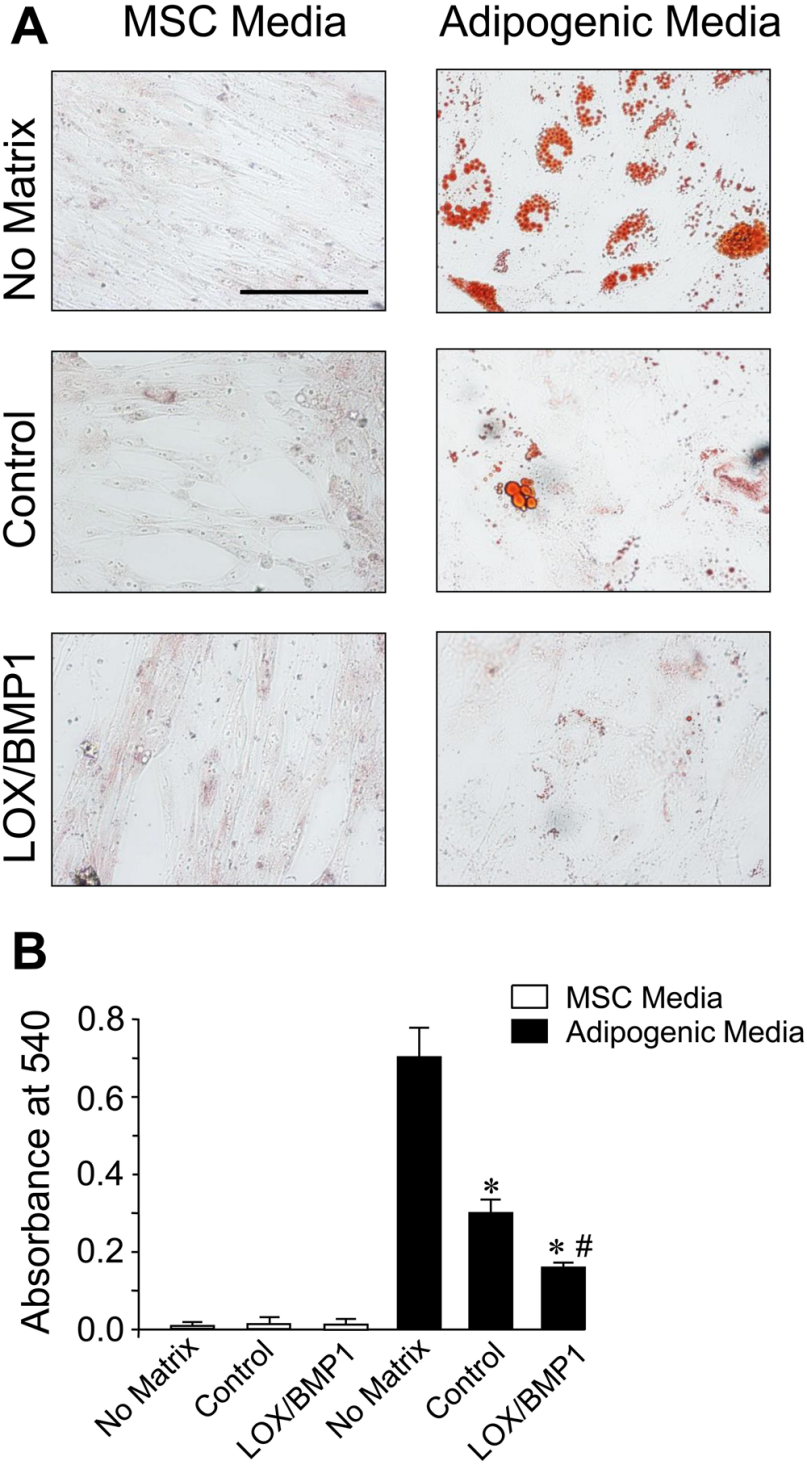
Adipogenic differentiation of human MSC seeded on decellularized matrices from fibroblasts exposed to LOX/BMP1 supernatants. Adipogenic capacity was evaluated by microscopic examination (**A**) and quantified by spectrophotometric analysis (**B**) using Oil Red O staining in human MSC seeded without matrix, with matrix from TGF-β-stimulated fibroblasts exposed to control medium or with LOX/BMP1. Micrographs shown correspond to representative results of staining performed twice with two independent preparations. Values are represented as absorbance at 540 nm (mean ± SEM, n = 6; *P < 0.05 vs no matrix, and ^#^P < 0.05 vs matrix fibroblast-derived matrix under control medium). Bars = 50 μm.

**Figure 8:**
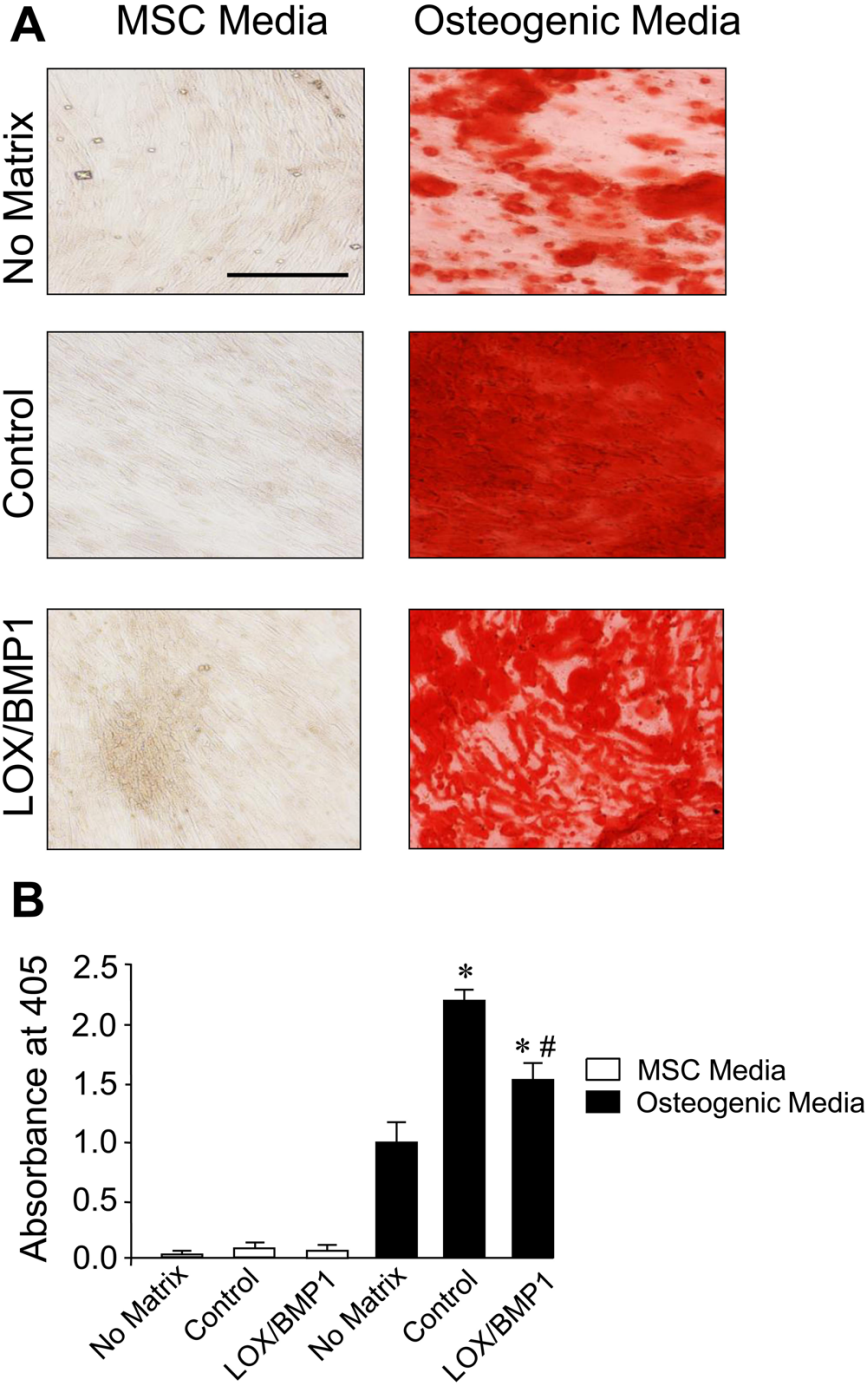
Osteogenic differentiation of human MSC seeded on decellularized matrices from fibroblasts exposed to LOX/BMP1 supernatants. The capacity of human MSC to differentiate into osteoblasts in substrates without matrix, with matrix from TGF-β-stimulated fibroblasts exposed to control medium or with LOX/BMP1 was assessed by microscopic examination (**A**) and quantified by spectrophotometry (**B**) using Alizarin Red S staining. Micrographs shown correspond to representative results of staining performed twice with two independent preparations. Values are represented as absorbance at 405 nm (mean ± SEM, n = 6; *P < 0.05 vs no matrix, and ^#^P < 0.05 vs matrix fibroblast-derived matrix under control medium). Bars = 50 μm.

Mesenchymal stem cells are a promising source for regenerative medicine due to its capacity to self-renew and to differentiate into various tissue lineages, such as adipocytes, osteoblasts, and chondrocytes. Since the ECM provides physical and chemical cues to regulate MSC activity, we investigated the effects of fibroblast-derived matrices modified by LOX/BMP1 on regulating MSC differentiation to adipogenic and osteogenic lineages. For that purpose, we exposed fibroblast cultures to control media or to LOX and BMP1-containing supernatants as described above, then cells were removed and deposited matrix used as a substrate to establish MSC cultures. Once these cultures reached confluence, they were induced into adipogenic and osteogenic lineages by incubation with the corresponding differentiation media. These cultures were then compared with equivalent MSC seeded without any matrix. As shown in Fig. 7, after 14 days under adipogenic differentiation medium MSC without matrix develop lipid droplets that can be visualized with Oil Red O staining. MSC cultured on matrices derived from fibroblasts exposed to control media showed a reduced capacity to differentiate to adipocytes, and this behavior was further exacerbated in matrices from fibroblasts incubated with LOX/BMP1. On the other hand, MSC differentiation into osteogenic lineage results in the formation of extracellular calcium deposits that can be specifically stained using Alizarin Red S, as shown in Fig. 8 for MSC without matrix. Osteogenic differentiation was strongly enhanced in MSC seeded on matrices from fibroblasts exposed to control media, this effect being attenuated in matrices from fibroblasts incubated with LOX/BMP1 supernatants. These results indicate that fibroblast-derived matrix is able to regulate adipogenic and osteogenic differentiation capacity of MSC, being the modification promoted by LOX/BMP1 capable to fine-tune this ability.

## Discussion

The biosynthesis of collagen is a highly complicated process involving numerous steps, including chain association and folding, secretion, procollagen processing and cross-linking^19^. Research on this pathway is relevant not only for mechanistic interest but also due to important technological applications. Nevertheless, in spite of a substantial knowledge of its molecular structure and the steps involved in the biosynthesis, the development of protocols to enhance the genuine capacity of cells to produce and deposit collagen *in vitro* is still at an early stage, mainly due to the intrinsic dilution limitations imposed by culture conditions. These constraints are translated into a deficient extracellular post-translational modification, basically at the level of the cleavage of the C propeptide, catalyzed by BMP1, and the formation of covalent cross-links, initiated by LOX enzymes^5,6^. Tackling the root of this limitation, here we describe that the implementation of fibroblast cultures with the enzymes BMP1 and LOX potently increased the capacity of the cells to deposit collagen onto an insoluble ECM. Analysis of the collagen intermediates representing the sequential steps in the biosynthetic pathway shows that LOX/BMP1 treatment increased pepsin-soluble and -insoluble fractions, both representing maturation products of collagen cross-linking. The ability of LOX/BMP1 to shift the biosynthetic pathway towards matured, cross-linked products was further corroborated by the significant, although modest, increase in the levels of the pyridinoline cross-links. One observation from our studies is the fact that the supplementation with LOX and/or BMP1 drastically reduced the accumulation of the soluble form of secreted collagen, but this reduction was translated to collagen deposition only when cells were concomitantly stimulated with TGF-β1. These findings suggest that, under our experimental conditions, the profibrotic effect of TGF-β1 is necessary to efficiently couple the collagen synthesis and secretion to the action of exogenously added LOX/BMP1. To this respect, it has been long known that TGF-β1 induces the expression of other enzymatic components contributing to collagen biosynthesis, including prolyl- and lysyl-hydroxylases, which in conjunction with the supplementation with LOX and/or BMP1 can potentiate collagen deposition^20,21^.

Our studies show that, among several matrix components, only collagen type I is significantly enhanced by the action of LOX/BMP1, an observation that is consistent with its prominent role in the ECM as primary substrate for these collagen-modifying enzymes^22,23^. While the accumulation of additional matrix components can not be ruled out, our observations suggest that collagen type I is the main component responsible for the biological effects of the LOX/BMP1-derived matrix, as analyzed upon decellularization and seeding of human MSC. Using these decellularized matrices, we found that those obtained from fibroblasts incubated under control conditions strongly inhibited adipogenic differentiation of MSC, and this effect was further potentiated in LOX/BMP1-derived matrices. To this respect, previously published reports provide also evidences showing that collagen type I exerts indeed an inhibitory role on adipogenesis. For instance, bone marrow-derived MSC cultured on collagen type I-coated plates were reported to display lower capacity to differentiate into adipocytes compared to cells on plastic^24^. On the contrary, diminished collagen type I production by MSC, as observed in the absence of ascorbate or in cells isolated from osteoporotic patients, potentiated adipogenic differentiation^25,26^. On the other hand, our decellularized matrices were found to enhance osteogenic differentiation, with LOX/BMP1 reducing this effect. While it has been previously observed that fibroblast-derived matrices amplify the osteogenic differentiation of MSC, and this effect can be attributed to collagen type I^24,27–29^, at this time, we can only speculate about a potential dual role for the effect of a collagen type I matrix on osteogenesis, promoting the differentiation at moderate levels as those observed in a control matrix, while inhibiting under LOX/BMP1 supplementation. Additionally, it must be also taken into account that the incubation with LOX and BMP1 might provide not only quantitative but also qualitative cues to the generated matrix, thereby regulating the propensity of cells to undergo lineage differentiation, as previously reported for native versus denatured collagen I matrix^30^. To this respect, the influence of an increased collagen cross-linking should not be underestimated, based on the inhibitory effect on osteogenesis of chemically-crosslinked collagen-glycosaminoglycan scaffolds as opposed to the same substrate in the absence of cross-linking^31^.

Cell-sheet tissue engineering exploits the inherent capacity of cells to create ECM scaffolds that, theoretically, should fulfill the structural and functional requirements for better preservation and growing conditions of MSC. However, because of the limitations imposed by cell culture environments, these technologies have not been efficiently translated to the clinical setting. An important breakthrough in the development of strategies specifically dealing with this issue has been the realization that the addition of so-called macromolecular “crowders” such as dextran sulfate strongly potentiates the capacity of a number of different cell types to deposit abundant matrix^5,9^. The use of this approach has led to a substantial reduction in the time required to create ECM-rich tissue equivalents^14,32^. Our experiments were performed with the addition of dextran sulfate. Therefore, on top of the benefits provided by macromolecular crowding, the supplementation with LOX/BMP1 has proved to further increase the capacity of the cells to deposit collagen onto an insoluble ECM. While the present study still represents the proof-of-concept to test and validate this protocol, it indeed opens the door to its application in the fabrication of implantable devices. Previously described cell-assembled systems for different tissues and organs may therefore take advantage of the use of our approach to accelerate the time required for *ex vivo* culture^33–39^. In a simple setting, our technology can be combined with the use of temperature-responsive plates, allowing the detachment of cell sheets by a temperature switch as already tested for macromolecular “crowders”^36,40,41^.

While the present approach present advantages over protocols previously established to enhance ECM deposition from cell cultures, it still has limitations or needs further validation in some aspects. For example, based on the analysis of the different collagen fractions, our study revealed that the fraction most robustly favored by the addition of LOX/BMP1 was that solubilized by pepsin, corresponding to recently cross-linked collagen, compared to the pepsin-insoluble fraction, which reflects more heavily cross-linked maturation products. This finding indicates that even in conditions favoring collagen deposition, progression to advanced maturation products is still limited. On the other hand, our approach relies on the supplementation of culture media with two enzymatic components that needs to be recombinantly produced, a circumstance imposing constraints to its technical development and commercialization. Nevertheless, in support of its use, their benefits were observed without the need to purify LOX and BMP1 from the corresponding cell supernatants. Finally, an important consideration in cell-based therapies is the potential to develop 3D-assemblies, an aspect particularly important in chondrocyte differentiation for cartilage matrix reconstruction. While proved to be suitable for adipogenic and osteogenic differentiation of MSC monolayers, our technology needs still to be validated in tridimensional MSC chondrogenesis. Nevertheless, this analysis is beyond the initial objectives of the present study.

## Methods

### Fibroblast cell culture

The human fibroblast cell line CCD-19Lu (ATCC) was maintained in culture medium as already described^42^. For collagen analysis, fibroblasts were seeded on 100-mm dishes in culture medium without serum and phenol red but containing 100 μg/ml 500 KDa dextran sulfate (DxS) and 29 μg/ml L-ascorbic acid 2-phosphate (Sigma-Aldrich, St. Louis, MO), for up to four days in the absence or presence of 5 ng/ml TGF-β1 (R&D Systems, Minneapolis, MN).

### Collagen analysis

Several fractions of collagen were extracted from the cultures representing the sequential steps in the biosynthetic process^43^. Cell supernatants were assayed for the soluble form of secreted collagen upon concentration with Sircol Soluble Collagen Assay (Biocolor, Carrickfergus, United Kingdom) following manufacturer’s instructions. Cell layers were scrapped, extracted overnight with acid-based buffer (0.5 M acetic acid), and resulting pellets digested with 0.5 mg/ml pepsin (Sigma-Aldrich) in 10 mM HCl. Corresponding acid- and pepsin-solubilized fractions, that represent recently deposited, non-crosslinked and recently cross-linked collagen, respectively, were also analyzed with Sircol. Heavily-crosslinked collagen from the insoluble fraction after pepsin digestion was hydrolyzed at 100 °C for 16 hours with 12 M HCl, neutralized with NaOH and analyzed by hydroxyproline assay using hydrolyzed type I collagen as standard^44^. Hydrolyzed fractions were also assayed for the content of the pyridinoline cross-links (PYD) using a commercially available ELISA kit (Quidel, Athens, OH).

Soluble collagen in the supernatant was also analyzed by western blotting using an specific anti-collagen α1 type I antibody (sc-8784, Santa Cruz, Dallas, Texas) upon protein fractionation in sodium dodecyl sulphate-polyacrylamide gel electrophoresis (SDS-PAGE) following protocols previously described^45^.

### Generation of HEK293 cell clones overexpressing LOX and BMP1

A full-length human LOX construct in pYX-Asc vector was obtained from Imagenes GmbH (Berlin, Germany). A full-length human BMP1 construct in pBabe vector was kindly provided by Víctor L. Ruiz-Pérez (Instituto de Investigaciones Biomédicas “Alberto Sols”, Madrid, Spain)^46^. Both constructs were cloned into the vector pcDNA5/FRT/TO (Invitrogen, Carlsbad, CA), to obtain the corresponding pcDNA5/FRT/TO-LOX and –BMP1 plasmids. These constructs were then co-transfected with the Flp recombinase expression plasmid pOG44 into the Flp-In T-REx 293 cell line using Lipofectamine 2000 (Invitrogen). These cells stably express the Tet repressor and contain a single integrated FRT (Flp recombination target) site. Flp recombinase expression from the pOG44 vector mediates insertion of the cDNA cassettes into the genome at the integrated FRT site through site-specific DNA recombination. After 48 hours, cells were selected for hygromycin B resistance (Roche Diagnostics, Barcelona, Spain), and clones appeared after 10–15 days. Isogenic pooled clones were expanded and checked for transgene expression after 48 hours of incubation in the absence or presence of the tetracycline analog, doxycycline at 10 μM. Culture media were concentrated using Amicon Ultra-4 centrifugal filter units (Ultra-Cel 10 K, Millipore, Cork, Ireland). LOX and BMP1 protein levels in cell layers or concentrated supernatants were detected by western blotting using specific antibodies against LOX (ab31238, Abcam, Cambridge, United Kingdom) and BMP1 (AF1927, R&D Systems). LOX enzymatic activity was determined using a commercially available assay from Abcam.

### Immunofluorescence studies

Fluorescence microscopy was performed as previously described^47^. Briefly, cells were seeded onto 10 mm glass diameter coverslips (No. 1.5) in 35 mm culture dishes (Mattek, Ashland, MA). After the corresponding treatment, cells were fixed with cold methanol for 5 min, blocked with 1% BSA in phosphate-buffered solution (PBS) for 1 h, and then incubated overnight at 4 °C with anti-collagen α1 type I antibody (Santa Cruz), anti-collagen IV (20421, Novotec, Lyon, France), collagen α1 type III (sc271249, Santa Cruz), anti-fibronectin (sc8422, Santa Cruz) and LOX (ab174316, Abcam), followed by the corresponding fluorescent secondary antibodies. Cell fluorescence was visualized by microscopy with a Nikon Eclipse T2000U (Nikon, Amstelveen, The Netherlands).

For analysis of the matrix deposited from cells, decellularization was performed by incubation with an extraction buffer containing 0.5% (v/v) Triton X-100 and 20 mM NH4OH in PBS for 3–5 minutes as previously described^48^.

### Analysis of adipogenic and osteogenic differentiation of human mesenchymal stem cells

Human mesenchymal stem cells (MSC) (Promocell, Heidelberg, Germany) were maintained in culture under basal medium (Promocell) and then induced for adipogenesis and osteogenesis with corresponding differentiation media (Promocell) for 14 days with medium change every 2–3 days. Phenotypic changes induced by lineage differentiation, i.e. the formation of lipid vesicles for adipogenesis and the extracellular deposition of calcium phosphate for osteogenesis, were monitored by staining with Oil Red O and Alizarin Red S (Santa Cruz), respectively, as described previously^49^. Differentiation was assessed by microscopic examination and quantitatively determined by spectrophotometric analysis upon dye solubilization.

### Statistical Analysis

Experimental data were analyzed using the unpaired Student t test in the case of normal distribution of data or using nonparametric tests as appropriate. The *P* values obtained are indicated in the figure legends when statistically significant (*P* < 0.05).

### Data Availability

The datasets generated during and/or analysed during the current study are available from the corresponding author on reasonable request.

## Electronic supplementary material

Supplementary Information
