# Supplementary material for: Enhancement of collagen deposition and cross-linking by coupling lysyl oxidase with bone morphogenetic protein-1 and its application in tissue engineering
Source: Sci Rep. 2018 Jul 17;8:10780. doi: 10.1038/s41598-018-29236-6 (PMC6050231; doi:10.1038/s41598-018-29236-6)
Supplement: Supplementary file 1 — Supplementary Information [file 41598_2018_29236_MOESM1_ESM.pdf]

Revised version of manuscript SREP-18-00860

Enhancement of collagen deposition and cross-linking by coupling lysyl oxidase with bone morphogenetic protein-1 and its application in tissue engineering

Rosell-García T, Rodriguez-Pascual F*.

Centro de Biología Molecular "Severo Ochoa" Consejo Superior de Investigaciones Científicas (C.S.I.C.)/ Universidad Autónoma de Madrid (Madrid), Madrid, Spain.

Supplementary Material

Supplementary Figures 1 and 2

Supplementary Fig. 1. Collagen type I immunoreactivity in the supernatants of fibroblast cultures supplemented with LOX- and BMP1-containing conditioned media. Fibroblast cultures were exposed to control or LOX/BMP1 supernatants in the presence (T) or absence (B) of TGF-β1 for 4 days and collagen type I immunoreactivity assessed by western blotting as described under Materials and Methods. Specific collagen type I immunoreactivity was detected as a TGF-β1-induced band of approximately 150 KDa. The blots shown correspond to representative experiments performed twice with two independent preparations.

Supplementary Fig. 2. Analysis of the immunoreactivity of collagen types IV and III, fibronectin and LOX in the decellularized matrices from fibroblasts exposed to LOX/BMP1 supernatants. Fibroblast monolayers exposed to control or LOX/BMP1 supernatants in the presence of TGF-β1 for 4 days were decellularized before processing for immunofluorescence analysis of collagen types IV and III, fibronectin and LOX as described under Materials and Methods. For comparison, immunofluorescence experiments done with control fibroblasts without decellularization are also shown in bottom panels (w/o decell.). Micrographs correspond to representative staining results performed twice with two independent preparations. Bars = 50 μm. DAPI staining was performed to confirm cell removal (not shown).

Supplementary Figure 1

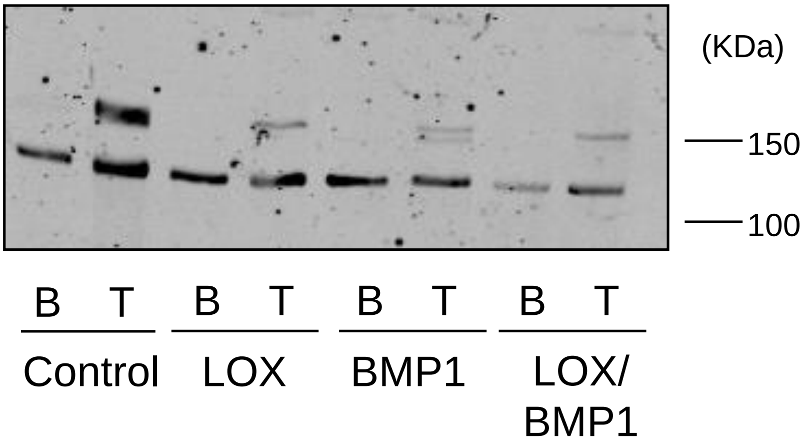

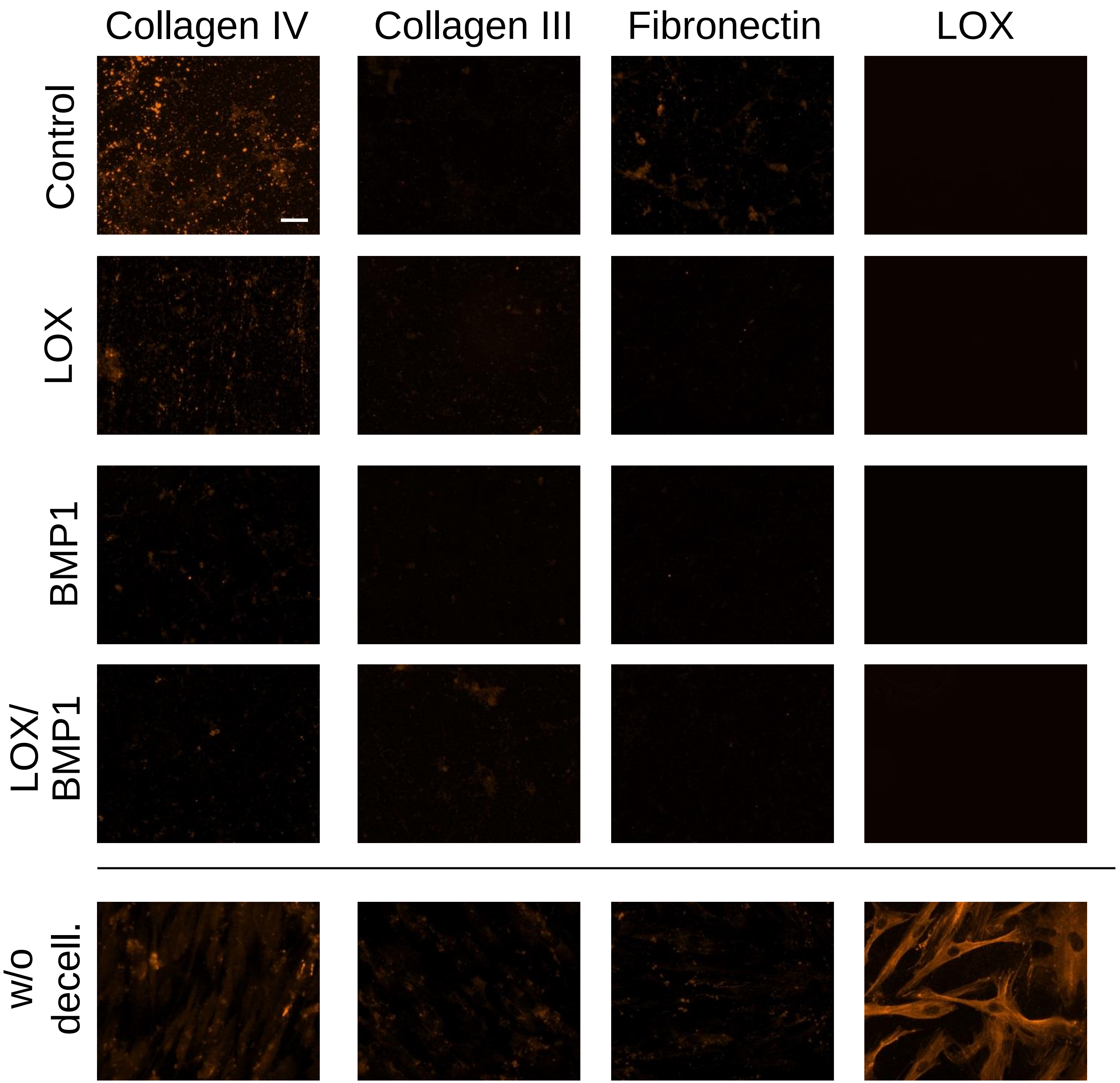

Supplementary Figure 2
